# Patient pathways in primary health care – an interview study across various health care personnel in a Canadian and a Norwegian county

**DOI:** 10.1186/s12913-024-11985-y

**Published:** 2024-11-28

**Authors:** Vivian Nystrøm, Arto Ohinmaa, Ann-Chatrin Linqvist Leonardsen

**Affiliations:** 1 Department of Health, Welfare and Organisation. Postal Box Code (PB) 700, Østfold University College, Halden, 1757 Norway; 2 School of Public Health, University of Alberta, 11405 - 87 Avenue, Edmonton, T6G 1C9 Canada; 3 Østfold Hospital Trust, Postal box code 300, 1714 Grålum, Norway

**Keywords:** Continuity, Coordination, Health care services, Interviews, Primary care

## Abstract

**Background:**

Due to demographic changes in the Western world governments emphasize the need for viable solutions, e.g. through decentralization of specialist health care services and better coordination within and between health care services. Both Norway and Canada have been through health care reforms and initiatives aiming to improve continuity and coordination of services. Organizational change to primary care in both countries encompasses both team-based service delivery involving allied health professionals, and new blended payment models. The objective of this study was to explore patient pathways in primary healthcare from various health personnel’s perspectives, and across various primary care organizations in Norway and Canada.

**Methods:**

The study had a qualitative design, including interviews with physicians, nurses and managers (*n* = 19) in primary care, from a county in Norway and a region in Canada. Data were analyzed with a thematic approach, in line with recommendations from Braun & Clarke.

**Results:**

Three themes were identified: 1) Structural challenges, 2) Towards a more specialized primary health care and 3) Dedication could improve continuity. Findings indicate that coordinating health care services was assumed difficult due to different health care levels, funding systems, managements, electronic record systems and organizations. Hospitals were assumed more task oriented, while primary health care services were considered more care oriented, and this challenged the coordination across organizations. Primary care services were perceived to be more and more specialized, also representing a threat for coordination and continuity. Health care personnel in both countries perceived that dedicated personnel for each patient could improve information flow and continuity across services.

**Conclusions:**

Achieving continuity and coordination of health care services seems challenging. Integration strategies seem essential for reducing silo thinking and fragmentation of health care services.

**Supplementary Information:**

The online version contains supplementary material available at 10.1186/s12913-024-11985-y.

## Introduction

Internationally, hospitals are over-crowded, and consequently hospital length of stay is decreasing [1]. In addition, there are not enough health care professionals to meet the demand for health care services [2, 3]. Hence, governments emphasize the need for finding viable solutions, e.g. through decentralization of specialist health care services and better coordination within and between health care services [4, 5]. Central public policy documents and research strategies highlight the need for pathways characterized by good quality and safe care, and which are based on user involvement, continuity of care and successful collaboration within and between service levels [6–8]. Several models have been developed, aiming to ensure coordination of services across primary and specialist health care, for example integrated care units, community hospitals and nurse-led units [9–11]. Studies indicate that such units increase continuity and coordination of health care, and thereby improve patient experiences [12–15].

Both in Norway and in Canada, health care systems have been through several reforms focusing on decentralization of services from specialist to primary care. Both countries are organized in various health care levels, defined as specialist or primary care. In Norway, the health care system is organized into two different governmental levels, each with different funding systems, laws, central regulations, and electronic patient journal systems [6]. Both hospitals and ambulance services are part of the specialist health care service. In many cases, treatment starts in the ambulance on the way to the hospital. For both somatic and physical conditions that require specialist treatment, patients are referred to a hospital. Municipal acute wards (MAWs), after-hour services, short- and long-term care, home care, and nursing homes are primary health care services, organized within the municipalities (*n* = 356). A primary care physician is the patient's most important and often the first point of contact with the health care service. They have a responsibility to coordinate the patient's health care services. Every resident has the right to receive a primary care physician who holds the medical responsibility for the patient [16]. To ensure transfer of sufficient and relevant information between hospitals and primary care, electronic dialogue messages have been introduced as a tool to improve collaboration. The dialogue messages comprize a set of standardized messages to support the admission, treatment, and discharge phases of a hospital stay [17].

The Canadian health care service is dived into 13 provincial and territorial tax funded insurance plans, each responsible for managing and organizing the universal health care coverage for their residents. The Canadian health care system consists of three levels. Medicare covers the specialist level including hospitals, physicians and diagnostics, based on national laws, regulations and a publicly funded health care system [18, 19]. Level two includes a combination of public, private funded and out-of-pocket payed primary care services, and covers prescription drugs, home care, long-term care and mental health care. Level three is private funded primary care services, covering Community Health Care, dental care, vision care, complementary medicine, and outpatient physiotherapy, all with limited public regulations [18, 19]. Hospitals, health authorities, and other organizations typically have their own boards and budgets, allowing them to make independent decisions about the services they offer [20]. National reforms have largely emphasized improving health care quality through forming primary care teams, creating partnerships, and establishing Primary Care Networks (PCNs), family health teams, and federations of physicians [20].

Health care reforms both in Norway and Canada focus on increasing continuity and coordination within and between health care personnel and health care organizations. Several studies have found that cooperation between caregivers to achieve continuity of care is deficient [21–23]. Many frail old persons require long recovery periods after discharge from hospital [24, 25]. A systematic review showed that frail older persons experience challenges due to abrupt discontinuation of health care after discharge from a hospital stay [26]. A study from Canada identified challenges of implementing comprehensive cancer care due to poor electronic systems that hindered communication and information transfer between primary care physicians and oncology specialists, and a lack of evaluation protocols that made it difficult to assess initiatives’ effects on patients, providers, and system outcomes [27]. Further, Canadian family doctors have reported a lack of routine communication with patient’s case managers or home care providers. In addition, they have reported difficulties coordinating care with social services or other community providers [28]. A register data study from Norway evaluated the continuity of care from 2006–2021, and the results showed large geographical variations [29].

Continuity and coordination of care involves a range of health care services, and effective care coordination requires addressing issues at an individual, organizational, and system level [30]. Relational continuity between health care professionals and especially older people have been shown to increase patient centered care and to reduce expenses [31, 32]. A systematic review found that relational continuity with a primary care physician whilst in hospital, can decrease hospitalization rates and emergency department visits in aged care [31]. In addition, nurse-led services offering coordinated care for chronic disease in both primary and secondary health care are associated with reduced hospitalizations and higher patient satisfaction [32].

Both Norway and Canada have been through several health care reforms and initiatives aiming to improve continuity and coordination of services. Organizational change to primary care in both countries encompasses both team-based service delivery involving allied health professionals, and new blended payment models [33]. The objective of this study was to explore patient pathways in primary health care from various health personnel’s perspectives, and across various primary care organizations in the two countries, to explore whether decentralization and reorganization of healthcare services may have impacted aspects such as coordination and continuity.

## Methods

This cross-country study had a qualitative design, utilizing individual, semi-structured interviews. The study adheres to the Consolidated Criteria for Reporting Qualitative Research (COREQ) guidelines [34].

### Setting and participants

In Norway, the study was performed in a county with approximately 320 000 residents. Primary health care services included after hour services, MAWs, and a municipal rehabilitation ward. In Canada, the study was conducted in a province with approximately 1,5 million residents. Primary health care services included community health care, PCN and home care. These wards were selected due to performing both acute assessments, following up on chronic diseases, and coordinating care with specialist health services, and were deemed appropriate for the study objective.

We used both a purposive and snowballing sampling method, selecting information rich cases for in depth-study. Both physicians, nurses, and managers were recruited (see Table 1). Inclusion criterion was experience from their current position of at least one year. In Norway, the first author sent an email directly to the participants, based on being familiar with the health care organizations. In Canada, all participants received information about the study and an invitation to participate by an email forwarded by the managers. All invited participants consented to participate, but one physician in Canada refused to participate. After accepting the invitation, no participants withdrew their consent to participate.

**Table 1:** Information on the study participants’ country, gender, categorized age in years, profession, and which primary health care service they worked in (*N* = 19)

Number	Gender	Age	Profession	Health care service
P 1C	F	50’	Manager	CHC/PCN
P 2C	F	60’	Nurse	CHC/PCN
P 3C	F	30’	Physician	CHC/PCN
P 4C	F	20’	Manager	CHC/PCN
P 5C	F	30’	Physician	CHC/PCN
P 6C	F	30’	Nurse	CHC/PCN
P 7C	F	50’	Manager	CHC/PCN
P 8C	F	30	Manager	HC
P 9C	F	40’	Nurse	HC
P 1N	M	30’	Physician	AHS
P 2N	F	50’	Manager	MAW
P 3N	M	40’	Physician	MRW
P 4N	F	50’	Nurse	MAW
P 5N	F	40’	Nurse	MAW
P 6N	F	30’	Manager	PCN
P 7N	F	40’	Manager	MAW
P 8N	F	40’	Manager	MAW
P 9N	F	30’	Nurse	AHS
P 10N	F	60’	Manager	AHS

*Abbreviations*: *P *Participant, *C *Canada, *N *Norway, *F *Female, *M *Male, *CHC *Community Health Care, *HC *Home Care, *AHS *After hour service, *MAW * Municipal Acute Ward, *MRW *Municipal rehabilitation ward, *PCN * Primary Care Network. Age is categorized in years of ten to ensure anonymity

### Data collection

A semi-structured interview guide focusing on issues related to continuity, coordination and patient centredness (appendix 1) was developed based on previous literature and through iterative discussions between the authors [35, 36]. Interviews were conducted until information power was reached [37], meaning that no themes were identified in further interviews. In Canada, face-to-face interviews were conducted by the first author in the participants’ offices from March to April 2022, and three additional interviews were conducted digitally in June 2022. In Norway, interviews were conducted at the participants’ workplace from April to June 2023, and three interviews were conducted digitally in June 2023. The interviews lasted from 21 to 54 min, with an average duration of 38 min. All interviews were audio recorded and stored on an encrypted UBS stick in a locked safe at the first authors office. All the material was transcribed verbatim by two external transcribers, one native English and one native Norwegian speaker respectively, who had signed a non-disclosure agreement.

### Analysis

Data were analyzed using thematic analysis as recommended by Braun and Clarke [37]. This method is described as a theoretically flexible approach to qualitative data analysis, that facilitates the identification and analysis of patterns or themes across data.

The interview transcripts were inductively analyzed using a six-step process:

1) In step 1, both first and last author familiarized with the data through listening to the recordings and then reading and re-reading the transcripts. 2) In step 2, both the first and last author inductively coded the transcripts, line-by-line. Any central quotes or key words were highlighted as codes. Based on the codes, initial themes were created, capturing important aspects about the data in relation to the study objective. The initial themes were then discussed between the two authors until agreement was reached. 3) In step 3, all authors reviewed the initial themes, across all interviews. The second author was involved via email, and in virtual meetings. 4) In the fourth step the themes were reviewed. Some themes were merged, and some were divided. The first and last authors went back and forth between the transcribed material and the subthemes and themes, to ensure the content and totality of the data. 5) Next, the final themes and subthemes were named. 6) In the last step an analysis report was written. Table 2 gives an example of the analysis process.

**Table 2:** Example of the thematic analysis according to Braun and Clark

Transcript	Codes	Subtheme	Theme
But for things to… continuity and coordination between the different health care services …to work, then there must be good information channels, there must be systems talking to each other….information must be easily accessible (Physician, N3)	continuity coordination between different health care services must be good information channels systems talk to each other information easily accessible	Fragmented and not seamless communication A need for access to complete and up-to date medical records	Structural challenges
..there is not a lot of communication… sometimes I don’t get that (discharge note) and that is limiting so you wasting time waiting to find out. And sometimes the patient will have seen someone and say the doctor told me I need to ask you for this. And if I don’t have that, we are going in circles until I know what went on. I think that is limiting from a continuity perspective (Physician, C3)	not lot communication sometimes I don’t get discharge note limiting wasting time patient have seen someone doctor told me need to ask for this going in circles until know what went on limiting continuity	Fragmented and not seamless communication A need for access to complete and up-to date medical records	Structural challenges

*N3* Participant number 3 from Norway, *C3 * Participant number 3 from Canada

## Results

In total 19 health care personnel participated in the study between April 2022 and June 2023. The participants’ age ranged from 27 to 54 years (median 42 years). We did not identify any variations in perspectives across professional groups or health care settings. Three themes were identified in the analysis: “Structural challenges”, “Towards a more specialized primary health care” and “Dedication could improve continuity”.

Quotes are marked with participant (P), number, *N* = Norway or C = Canada, and professional background (manager, physician, nurse).

### Structural challenges

All participants from both countries perceived a challenge due to the health care service being structured within a hospital level and a primary health care level. This two-tiered structure represented different funding system, different management, and different institutions. In the participants’ view, and across countries, hospitals were more directed toward tasks, including diagnostics and medical treatments, while primary health care was considered more care oriented approaching the patient more holistic. This was described to limit the coordination of services across the levels. P 1N, a physician, described this as follows*: «…some factors that limit it… we are different entities with different budgets, different management, and there is literary a wall between primary care and the hospitals.”*

This was supported by P 7C, manager, who stated: *“We are funded differently and employed differently [meaning: hospitals and primary care]”.*

Across all of the participants, the communication flow between health care levels were described to be fragmented and not seamless, as intended. This was related to the different documentation systems, and the various oral communication flow. In Canada, participants referred to the system Netcare ®, which was reported to be well suited to primary health care. The similar documentation system in Norway was referred to as Gerica ®. However, neither of these two documentation systems communicated digitally with the documentation system used in hospital. Having access to complete and up-to-date medical records was highlighted by most of the clinicians as a key to coordinate the health care services, and hence this was experienced as a limitation. The documentation systems were assumed to minimize the need for redundant data entry and to save time, in addition to facilitate making informed decisions about patient care, however this did not work as intended when systems across health service levels differed. P 3C, physician said: *“In general, when I send patients out to specialists, I get a note back within a week or so saying this is what we have done. Sometimes I don’t get that and that is limiting so you’re wasting time waiting to find out.”*

This was also described by e.g. P 5N, nurse: *“Inadequate communication is the correct term to use. If we used the same tools, we would understand both sides of the issue more clearly.”*

The lack of continuity was also related to oral communication between hospital and primary health care, which was described to be both challenging and time consuming. P 9C, nurse described it like this: *“From my perspective it is harder to get a hold of hospital staff because they work different shifts, and they go through different units. They don’t keep a cell phone on them. You basically have to call units and say..can you find… are they there…”.*

This was supported by P 3N, physician: *“Then you call this number, and you will get ten different options within these subspecialties of internal medicine. Generally, there is someone on the other end of the line, but not always.”*

Across countries, participants also underlined the challenges in coordinating health services after discharge from hospital. In Canada, the discharge process from hospital to home was reported to be coordinated by a “transition coordinator” and in Norway the “tildelingskontor” (provision office). The participants described these to be important to ensure a smooth transition of care between specialist and primary health care. This also included assessing and advocating the patients’ needs and coordinating the communication between the patient and the health care providers. Nevertheless, challenges with the discharge process were frequently discussed by many participants in both countries. P 6N, manager reported:

*“And I don't think that we in the primary care are good enough at, in a way, truly understanding what the patient's needs are. Because we are under pressure to accept patients, regardless of what the needs actually are… I see that we are behind from the moment we receive patients, meaning the notification of admission from the hospital.”*

P 7C, manager, supported this, stating: *«If we are lucky enough to know that the senior has been discharged from the hospital. If that patient is one of our paneled patients, then if they are frail enough or suffering from dementia, our geriatric program has most likely been involved to some degree. But it is not a direct link.”*

### Towards a more specialized primary health care

All participants, regardless of profession, health care setting or country, described that primary health care services seemed to be more and more specialized. Both in Norway and Canada, participants referred to that patients either had a general practitioner (Norway) or a dedicated physician (Canada). Furthermore, in Norway participants described more specialized units like “municipal acute wards”, “rehabilitation units”, “palliative units”, “short time units”, and “long time units”. In Canada, participants described the services organized in centralized programs like low maternity clinics, nutrition programs, health education programs, diabetes programs, health exercise programs and pain management programs. Also, clinics in primary health care could be staffed by licensed practical nurses, registered nurses, podiatrists, pharmacists, obstetricians, or gynecologists. A manager described the PCN as a service supporting the nursing homes. However, the services provided by PCNs were more targeted treatment interventions rather than comprehensive care. One of the managers in Norway stated that a patient bed should be a patient bed without needing to assess the appropriateness of the bed against the patients’ condition. P 6N, manager, meant that such specialization was a threat for coordination and continuity of health care, and stated: *“I am a little bit sceptical because we have the target group of patients that we have and I am a little unsure whether it is right that the municipalities become so specialized because there are so many people who fall between our target goal of patients who need help.”*

However, more specialized primary health care was not assumed to be a threat for the continuity in the same way in Canada. This was illustrated by P 7C manager, who said:

*“If I am a newly diagnosed diabetic, I can expect to go into my new diabetic home and my labwork would have shown up to my physician and the physician would diagnose me as a new diabetic. And rather than getting into an hour and a half conversation of what that means to me, that physician would hand me off to a registered nurse. And that registered nurse would then discuss what that means to me and how I need to adjust my lifestyle and diet or if I were ready to make those changes.”*

However, several of the Norwegian managers reported a growing concern about patients being treated in a unit outside the range of services that the patient needed. In their opinion, this affected the quality of care provided, and also whether they were prioritized. P 6N, manager said: *“I have received a Non-Conformity Report with a concern that palliative and short-term patients should not be in the same unit. And then I think like that; wow – the patient’s needs help. Now you have to think about what you learnt under your education and try to continue the holistic care and nursing for all patients.”*

The participants in both countries also highlighted a large increase in the number of people with mental illness. Our current healthcare system lacks effective collaborative platforms necessary to adequately support users requiring multiple services or comprehensive care within primary healthcare settings P 3C, physician said:

*“Before, I would just see them for an ankle problem. Now, I see them for the ankle problem and there is always a number two, for they will tell me they are feeling stressed or overwhelmed, or feeling anxious and not sleeping well. I will say I see 30 patients a day, 10–15 are mental health patients.”*

This was supported by e.g. P 10N, nurse, who added: “*We simply lack effective collaborative platforms for users who require multiple services or various parts of our healthcare services in primary health care.*”

### Dedication could improve continuity

Across participants in both countries, there was a broad agreement that having a dedicated physician was important for the continuity of care, in a positive way. Regardless, if the primary care physicians were responsible for a group of patients, a panel, (in Canada), or list-based (in Norway), pitfalls like different health care levels and increasing specialization were described. P 1N, a physician from an after hour service in Norway, described that he was often the one who initiated meetings to coordinate health care service and to make plans for patients. P 10N, a nurse in Norway described that she experienced lack of engagement from the primary care physician and connected this lack of engagement to high workload. She meant there was a need to arrange cooperation arenas where different health care professionals could work together, illustrating it like this: *“I think we should agree about a common cooperation arena where we can discuss the more complex patients… nurses at the after hour services, home nursing, primary care physicians … we all have the same patient.”*

P 9C, nurse, pointed this out like this*: “To facilitate our services, to provide the most comprehensive care, our interdisciplinary teams are quite useful because we have a very specific area we work in, so we will repeatedly work with the same clients and our coworkers.”*

One of the most important issues to ensure continuity reported by all participants in both countries was to make sure that a discharge note from the hospital to the primary care was available. One of the managers described it like this: *“One important factor is a precis journal at the referral time. Why are the patients here? What are we going to treat? What must be done before the patient is medically clarified to be discharged back to home?”* (P 2N, Manager).

P 3C, physician also underlined: “*I would say the biggest continuity of care component is probably the electronic medical record. Internally we have access to each other’s notes. So, if I see a patient and they see Dr. X tomorrow or a few days later, he can see what I have done, which is good for continuity and chronic medical care.*”

Route of employment was also mentioned to be an important factor for the continuity. In Canada, it could be problematic when the physicians worked within different programs and if the patients were not paneled or attached to one specific physician. In Norway, part time employment and rapid staff turnover were assumed to be a challenge for continuity. One success factor that some of the participants reported was having the same personnel at the same unit over time. P 2N, a manager, expressed it in this way: *“Continuity with the same primary care physician following up to most extent is one of the most important factors, as well as continuity with the nurses and the license practical nurses.”*

Also, P 7C, a manager, stated: *“We do have a problem right now, and it is a system’s problem, where we have physicians retiring and we don’t have enough new physicians coming in to look after a panel.”*

## Discussion

To our knowledge, this is the first study exploring different health care personnel’s perspectives on patient pathways in primary health care- across various services and across two different countries. Results show that regardless of professional background, health care setting, or country, several issues were similar. Here, coordination was assumed difficult due to the different health care levels, funding systems, managements, electronic record systems and organizations. Hospitals were assumed more task oriented focusing on specific diagnostics or medical treatments, while primary care services were considered more care oriented focusing more on person-centred aspects. This challenged the coordination across services. Moreover, primary health care services were perceived to be more and more specialized, also representing a threat for coordination and continuity. Health care personnel in both countries perceived that dedicating personnel to each patient could improve information flow and continuity across services.

One major barrier to continuity and coordination, as reported by the participants in the current study, was the fact that health care services were structured within two different levels: hospital and primary care. Similar challenges were underlined in a rapid review of studies focusing on priorities and challenges for health leadership and workforce management globally [38]. Here, findings showed that health care organizations require various professionals with different competencies to deliver high quality care. The authors concluded that a dominant hierarchical culture and lack of collaborative culture may limit the performance of health care organisations. As such, the two different levels, as seen in both Norway and Canada, are organized and funded differently. This affect how services are provided, based on the established culture at each level respectively [39–41]. This may for example have implications for transfers between levels [40, 42]. As underlined in our study, evidence show that inadequate transfer of information between different levels of healthcare poses a threat to patient safety [41]. Both increased hospital readmission rates and increased morbidity have been reported as direct consequences of inadequate transfer [43].

The communication between the different levels in our study was described as fragmented, rather than seamless as intended. In both countries, this was specifically related to different documentation systems. Access to complete and up-to-date medical records was highlighted by most clinicians as a key element in coordinating healthcare services, which is supported by the literature [41]. In addition, communication channels between the different levels were a challenge. It was described as difficult to establish contact, and this was something that healthcare professionals in both countries spent a lot of time on. This is supported by e.g. Smith et al., who found that physicians and pharmacists highlight a lack of time, poor communication with specialists, and fragmented care as barriers to effective care [44]. Indeed, poor communication within healthcare settings can have significant ramifications for patients [45]. The misperception or misinterpretation of information conveyed can contribute to misunderstandings, heightened anxiety levels, decreased patient satisfaction, and even give rise to formal complaints [46, 47].

In the current study, there was a clear trend towards a more specialized primary healthcare service in both countries. The increasing number of elderly patients with multiple illnesses requires different types of healthcare services even within primary health care. Even if our approach adds a new aspect to primary care research findings, this development was also underlined in a systematic review on decision making and outcomes for patients with complex care needs in primary care settings [48]. The phenomenon of fragmentation and silo thinking has been described for many years as a problem in hospitals [49]. A Danish study showed that the number of involved health care providers, provider transitions, and hospital trajectories rose with increasing morbidity levels. Patients with three versus six conditions had a mean of 4.0 versus 6.9 involved providers, and 6.6 versus 13.7 provider transitions [50]. This poses a threat to patient safety, and research has shown that high levels of care fragmentation are associated with increased rates of potentially inappropriate medication use and higher mortality rates [50].

In the current study, there was an understanding in all stakeholders in both countries that dedication could improve continuity of care. Specifically, having a dedicated physician was deemed important for the continuity of care. Research show that general practitioners express worries that they will not be able to provide the population with the expected care and services in the future [51, 52]. Previously, physicians had the responsibility of ensuring necessary medical treatment for patients. However, nowadays, physicians are also responsible for coordinating services within and between different health care facilities [16]. This increased workload necessitates the exploration of new methods for organizing health care services. As new health care models emerge, both team-based care and nurse-led care have shown to enhance continuity and coordination of health care services [30, 53, 54]. This is particularly crucial for patients in need of complex services, such as those with chronic illnesses or older individuals whose health literacy and ability to take responsibility for their own health may be reduced [30]. Therefore, the presence of a responsible stakeholder with an overview of necessary health care services may become the most significant factor in ensuring effective care.

### Strengths and limitations

The qualitative research design entails a lack of opportunity for generalization [37]. Although different models in primary care have been developed, the primary health care models appear to be similar in many other Western countries [14]. This supports the transferability of our findings. The inclusion of a variety of health care personnel across various services may also be seen a limitation. However, similar findings across countries, professions and health care settings support the dependability of our findings. Also, saturation was reached in each country, meaning that no new themes were identified in consecutive interviews.

The interview guide was not piloted, and this may bee seen as a limitation. However, the guide was based on previous literature, and the participants were also encouraged to elaborate upon issues. In Norway, there is a legal commitment, stated in all laws regulating health and social services, to write individual plans for service users with long lasting and complex service needs [55–57]. Such plans were not mentioned in the interviews. This may be due to not being included in the interview guide.

A strength of this study was that all interviews were written down verbatim, by native speaking transcribers, including both verbal and non-verbal utterances, ensuring the internal validity and consistency of the findings. All authors were involved in the discussion of codes, subthemes and themes, thereby achieving confirmation.

Credibility refers to confidence in the “truth” of the findings, which was achieved through the transparent description of the data collection and analysis [37]. The research group had a broad composition, representing both countries, different genders, educational backgrounds and roles. All had in-depth knowledge about the different countries health care systems respectively. This contributed to a broader interpretation of the analyses of the different stakeholders’ perceptions of coordination and continuity of care across countries. The presence of different genders reduces gender bias.

## Conclusion

Health care personnel expressed concerns related to health care services being organized in different levels, and an increasing specialization of primary care. More integrated care initiatives should be prioritized in further development of primary care. This includes complementary work at the micro, meso and macro: i.e. at the clinician level, at the organizational level and at the system level.

### Implications for practice

Authorities, politicians, and health care leaders should focus on decreasing “the gap” between health care levels and professionals when developing, implementing and refining health care services.

## Supplementary Information

Supplementary Material 1.

## Data Availability

Datasets generated and/or analyzed during the current study are not publicly available due to local ownership of data. Aggregated data are available from the corresponding author on reasonable request.
