# Supplementary material for: Patient pathways in primary health care – an interview study across various health care personnel in a Canadian and a Norwegian county
Source: BMC Health Serv Res. 2024 Nov 28;24:1494. doi: 10.1186/s12913-024-11985-y (PMC11603772; doi:10.1186/s12913-024-11985-y)
Supplement: Supplementary file 1 — Supplementary Material 1. [file 12913_2024_11985_MOESM1_ESM.docx]

| INTERVIEW GUIDE  Introduction: Thank you for participating. This is a collaborative study between researchers in Canada and in Norway. Hospitals are crowded, and national and international healthcare reforms emphasize the need for more efficient services, decentralization of specialist healthcare services and better coordination between healthcare services at all levels. Moreover, healthcare politics and services have been moving away from the “doctor-knows-best approach”, towards a focus on person-centeredness, to ensure that the patient is at the centre of care delivery and decision making. Norway and Canada have implemented reforms and innovative ways of organizing healthcare services, aiming to meet these issues.  Explanation in Canada: In Norway the healthcare system is organized within two different governmental levels. State-owned hospitals provide specialized medical services, while the municipalities (n=356) are responsible for organizing primary healthcare, short- and long-term care, home nursing, and nursing homes to their residents. Every inhabitant is listed with a primary care physician who hold the medical responsibility for patients on his or her list. Hospitals and primary healthcare are subject to different funding systems, laws and central regulations, and also to different electronic patient journal systems. From 2016 municipal acute wards were implemented, as alternatives to hospitalisation.  Explanation in Norway: In Canada, the provinces (n=10) and territories (n=3) are sub-national administrative divisions within the geographical areas of Canada under the jurisdiction of the Canadian Constitution. Canada's healthcare system consists of 13 provincial and territorial health insurance plans that provide universal health care coverage to Canadian citizens, permanent residents, and certain temporary residents. These systems are individually administered on a provincial or territorial basis, within guidelines set by the federal government. The reform initiatives implemented across the jurisdictions have largely emphasized quality improvement and incentive-base levers, and/or organizational changes to practice, including the formation of primary care teams, partnerships, networks, or federations of physicians.  Few studies have explored different stakeholders’ perspectives on issues such as continuity, coordination and patient-centredness across services. The objective of this study is to explore different stakeholders from different healthcare service organizations’ perspectives on aspects such as continuity, coordination and patient-centredness across these two countries.  **Background information:**  Age, educational background, experience from current position  **Questions/themes**  1. Can you please describe the services provided at your unit/ward/institution?  Follow up: Who are the patients? What diagnostic opportunities/treatments do you offer?  2. In your opinion, which factors facilitate continuity of care?  3. Which factors limit continuity of care?  4. In your opinion, which factors facilitate coordination of services?  5. Which factors limit coordination of services?  6. In your opinion, which factors facilitate patient-centred care?  7. In your opinion, which factors limit patient-centred care?  8. In your opinion, what could be done to improve continuity of care, coordination of services and patient-centred care?  **Finisher**   - Is there anything else regarding these healthcare services that you have had in mind, or that you wish to tell more about   **Norsk versjon**  1. Kan du beskrive tjenestene som tilbys ved din enhet/avdeling/institusjon?  Oppfølging: Hva slags type er pasienter behandler her? Hvilke diagnostiske muligheter/behandlinger tilbys her?  2. Hvilke faktorer mener du legger til rette for kontinuitet i omsorgen?  3. Hvilke faktorer begrenser kontinuiteten i omsorgen?  4. Hvilke faktorer mener du legger til rette for samordning av tjenester?  5. Hvilke faktorer begrenser koordinering av tjenester?  6. Hvilke faktorer etter mener du legger til rette for pasient medvirkning?  7. Hvilke faktorer begrenser pasient medvirkning?  8. Hva mener du kan gjøres for å forbedre kontinuiteten i omsorgen, koordinering av tjenester og pasientsentrert omsorg?  Avslutning:  • Er det noe annet angående disse helsetjenestene du har tenkt på, eller som du ønsker å fortelle mer om? |
| --- |
